# Elderly fall risk prediction using static posturography

**DOI:** 10.1371/journal.pone.0172398

**Published:** 2017-02-21

**Authors:** Jennifer Howcroft, Edward D. Lemaire, Jonathan Kofman, William E. McIlroy

**Affiliations:** 1 Department of Systems Design Engineering, University of Waterloo, Waterloo, Canada; 2 Centre for Rehabilitation Research and Development, Ottawa Hospital Research Institute, Ottawa, Canada; 3 University of Ottawa, Faculty of Medicine, Ottawa, Canada; 4 Department of Kinesiology, University of Waterloo, Waterloo, Canada; University of Florida, UNITED STATES

## Abstract

Maintaining and controlling postural balance is important for activities of daily living, with poor postural balance being predictive of future falls. This study investigated eyes open and eyes closed standing posturography with elderly adults to identify differences and determine appropriate outcome measure cut-off scores for prospective faller, single-faller, multi-faller, and non-faller classifications. 100 older adults (75.5 ± 6.7 years) stood quietly with eyes open and then eyes closed while Wii Balance Board data were collected. Range in anterior-posterior (AP) and medial-lateral (ML) center of pressure (CoP) motion; AP and ML CoP root mean square distance from mean (RMS); and AP, ML, and vector sum magnitude (VSM) CoP velocity were calculated. Romberg Quotients (RQ) were calculated for all parameters. Participants reported six-month fall history and six-month post-assessment fall occurrence. Groups were retrospective fallers (24), prospective all fallers (42), prospective fallers (22 single, 6 multiple), and prospective non-fallers (47). Non-faller RQ AP range and RQ AP RMS differed from prospective all fallers, fallers, and single fallers. Non-faller eyes closed AP velocity, eyes closed VSM velocity, RQ AP velocity, and RQ VSM velocity differed from multi-fallers. RQ calculations were particularly relevant for elderly fall risk assessments. Cut-off scores from Clinical Cut-off Score, ROC curves, and discriminant functions were clinically viable for multi-faller classification and provided better accuracy than single-faller classification. RQ AP range with cut-off score 1.64 could be used to screen for older people who may fall once. Prospective multi-faller classification with a discriminant function (-1.481 + 0.146 x *Eyes Closed AP Velocity*—0.114 x *Eyes Closed Vector Sum Magnitude Velocity*—2.027 x *RQ AP Velocity* + 2.877 x *RQ Vector Sum Magnitude Velocity*) and cut-off score 0.541 achieved an accuracy of 84.9% and is viable as a screening tool for older people at risk of multiple falls.

## Introduction

The ability to maintain and control postural balance is important for standing, walking, and activities of daily living [1]. In older adults, poor postural balance can indicate an impaired ability to recover from small postural perturbations [2] and be predictive of future falls [3,4]. Static posturography can assess passive postural control, which encompasses responses to gravity and effects of relatively small, self-initiated corrective movements [5].

Posturography tests use force plates to measure center of pressure (CoP) movements while the participant stands on the platform, often looking at a fixed point [4]. Test difficulty can be increased by removing visual input, referred to as eyes closed. The eyes closed condition increases postural sway in adults of any age, compared to eyes open [6]; however, differences between eyes open and eyes closed are typically greater for elderly than younger adults. While other sensors and measures can be used to assess posturography, some important force-platform-based sway measures include area, path length, path area, mean frequency, fractal dimension, total power, 50% power frequency, 95% power frequency, and centroidal frequency parameters of CoP displacements [7,8]. A large study with 7,979 participants (2730 over 60 years old) found increased anterior-posterior (AP) plantar CoP speed and velocity moment (mean area covered by the CoP movement per unit time) with eyes closed [3]; however, no statistical analysis was reported.

A review of force-platform-based static posturography studies identified four differences in posturography measures between elderly fallers and non-fallers and a need for additional studies based on prospective fall occurrence to better understand the predictive value of static posturography for fall risk [4]. Fallers exhibited greater medial-lateral (ML) sway amplitude with eyes open and closed, AP speeds with eyes open, ML CoP root mean square distance from mean (RMS) with eyes closed, and mean CoP speed with eyes closed conditions than non-fallers [4]. Since this review, additional posturography measures have been used to identify differences between elderly fallers and non-fallers: including, AP and ML sway mean, RMS, and range [9–11]; ratio of ML to AP sway range [12]; AP and ML sway velocity [10,11,13,14]; 95% confidence ellipse area [11]; sway coefficient [12]; AP and ML frequency parameters [10,12,13,15]; detrended fluctuation analysis parameters [13]; AP and ML power spectral density parameters [10]; and stabilogram-diffusion analysis parameters [9]. Maki et al. [16] identified fallers with a false positive rate of 25% when positive prediction was 100%, based on deviations from normative posturography data using cross-spectral analysis, least squares, and maximum likelihood classification methods. Topper et al. [17] used posturography and logistic regression to identify fallers and non-fallers and achieved an accuracy of 65%, sensitivity of 78%, and specificity of 46%. Brauer et al. [18] used posturography and logistic regression to identify fallers and non-fallers with a sensitivity of 29% and specificity of 88%. Few studies have examined posturography measures for multi-fallers [11,19,20]. Stel et al. [19] found that increased ML sway was predictive of recurrent fallers, based on 1-year prospective fall history and Buatois et al. [20] reported greater sway with eyes closed for multi-fallers than non-fallers, based on 16-month prospective fall occurrence. Merlo et al. [11] found greater AP and ML RMS on a compliant surface for multi-fallers than single-fallers and non-fallers, based on 1-year retrospective fall occurrence; and a greater 95% confidence ellipse area for multi-fallers compared to non-fallers on a firm surface. Bigelow and Berme [13] used posturography, in combination with body mass index and age, and logistic regression to identify non-recurrent and recurrent fallers with 75% sensitivity and 94% specificity. It should be noted that, while the discussed studies used similar methods to define fallers and non-fallers, fall definitions were not identical; some studies used the Tinetti fall criteria [9,13,15,18,20], Kellogg fall criteria [14,19], CERAD assessment criteria [11], or ProFaNE fall criteria [12], and others did not specify specific fall criteria [10,16,17].

Romberg Quotient (RQ) could be used to identify posturography differences between fallers and non-fallers, but is not commonly used in posturography assessments. RQ is the ratio between eyes closed and eyes open values for a variable [21] and is potentially useful for identifying fallers because it measures an individual’s reliance on visual input for postural control. RQ quantifies the degree to which balance worsens (e.g., increased sway length or speed) when vision is removed, compared to an eyes open baseline.

This study investigated eyes open and eyes closed standing posturography with elderly adults to identify differences and determine appropriate outcome measure cut-off scores for prospective faller, multi-faller, and non-faller classifications. This information can be used as a screening tool for older people at risk of falling.

## Materials and methods

The methodology is summarized below, Details have been described previously in [22].

### Participants

A convenience sample of 100 people, aged 65 years or older, were recruited from local churches, retiree associations, and independent-living retirement homes. The University of Waterloo Research Ethics Committee approved the study and all participants gave informed written consent. Participants were excluded if they had a cognitive disorder or were unable to walk for six minutes without an assistive device, which was part of the larger study protocol. Exclusion due to cognitive disorder was based on participant self-report of medically diagnosed cognitive disorders.

Falls were defined as 'an event which results in a person coming to rest unintentionally on the ground or other lower level, not as a result of a major intrinsic event (such as a stroke) or overwhelming hazard' [23]. The 100 participants were divided into the following groups:

- Retrospective fallers (RF): 24 people who fell one or more times in the six-month period before data collection.
- Retrospective non-fallers (RNF): 76 people who had not fallen in the six-month period before data collection.

All 24 RF completed the six month fall follow-up. Of the 76 RNF, 75 completed the six month fall follow-up. The 99 participants with a complete prospective fall record were grouped as follows:

- Prospective all fallers (PAF): 42 people who fell during the follow-up period.
- Prospective faller (PF): 28 people who fell during the follow-up period, as a subset of the 75 RNF with a complete prospective fall record.
- Prospective non-faller (PNF): 47 people who did not fall during the follow-up period, as a subset of the 75 RNF with a complete prospective fall record.
- Prospective single-fallers (PSF): 22 people who fell once during the follow-up period, as a subset of the 28 PF.
- Prospective multi-fallers (PMF): 6 people who fell more than once during the follow-up period, as a subset of the 28 PF.

Table 1 reports anthropometric and demographic data for the participant sub-populations.

**Table 1:** Participant characteristics by fall group (mean ± standard deviation).

	n	Male/Female	Age (yrs)	Height (cm)	Weight (kg)
RF	24	13/11	76.3 ± 7.0	165.2 ± 10.3	71.9 ± 14.3
RNF	76	31/45	75.2 ± 6.6	165.1 ± 10.0	73.1 ± 13.4
PNF	47	17/30	75.3 ± 5.5	164.8 ± 10.5	73.3 ± 13.6
PAF	42	22/20	75.6 ± 7.8	165.7 ± 10.0	72.3 ± 13.5
PF	28	14/14	75.0 ± 8.2	165.7 ± 9.3	73.4 ± 13.2
PSF	22	11/11	75.9 ± 8.2	164.9 ± 8.9	69.9 ± 11.0
PMF	6	3/3	71.8 ± 8.1	168.7 ± 10.9	86.2 ± 13.2
All	100	44 / 56	75.5 ± 6.7	165.1 ± 10.0	72.8 ± 13.5

Separate analyses were performed for retrospective (RNF, RF) and prospective groups (PNF, PAF, PF, PSF, PMF) because a pre-assessment fall may cause a participant to develop fear of falling and change gait patterns [24]. Furthermore, RF would already be identified as being at increased risk of future falls due to their fall history [25].

### Data collection protocol

Participants reported six month retrospective fall occurrence, age, and sex. Body weight and height were measured.

Two Wii Balance Boards (WBB) were placed such that their long axes were oriented parallel to the AP axis, as described in [22]. Recent WBB studies reported good correspondence with force platform measures [26,27], excellent test-retest reliability for one-board [26,28] and two-board [29] configurations, and good to excellent concurrent validity [26,28] for CoP displacement measures. Participants stood in comfortable stance on two WBBs, with one foot on each board. Participants stood quietly with eyes open and then with eyes closed, while WBB data [30] were collected at 100 Hz for 30 seconds for each condition. Each condition was assessed once for each participant.

After completing the data collection session, participants were asked to record fall occurrence for the next six months on a calendar and fall information form (see S1 Fall Information Form). Participants were contacted monthly to collect fall information and ensure continued participation during the follow up period.

### Data processing

Vertical force data from the WBB were filtered using a 15 point moving average filter and plantar CoP were computed using purpose-built software (NiMBaL Balance Assessment, University of Waterloo). Outcome variables were AP and ML absolute CoP motion range (Range); AP and ML CoP RMS; mean AP and ML CoP total excursion velocities; and AP and ML mean resultant CoP velocity vector sum magnitude (VSM) [7]. RQ was calculated for all variables using Microsoft Excel.

### Data analysis

Normality was assessed for each variable using the Shapiro-Wilk test (α = 0.05). For eyes open and eyes closed comparisons, a paired *t*-test was used for normal variables and a Wilcoxon Signed-Rank Test was used for non-normal variables. For faller versus non-faller comparisons, a Mann-Whitney U Test was used for non-normal variables and a Levene Test for equality of variance was used for normal variables. An independent *t*-test was used for equal variance and a Welch’s *t*-test was used for unequal variance. Significance was tested at *p* < 0.05.

For variables that were significantly different between PF and PNF, or PMF and PNF, a cut-off score for faller classification was determined using the Clinical Cut-off Score [31], Receiver-Operator Characteristic (ROC) curves, and discriminant functions. Three different techniques were investigated to increase the likelihood that the strongest cut-off score was identified.

The clinical cut-off score, was calculated as:

$$Clinical\;Cut\text{-}off\;Score=\frac{\sigma_{n}\mu_{c}+\sigma_{c}\mu_{n}}{\sigma_{n}+\sigma_{c}},$$ (1)

where *σ*_*n*_ and *σ*_*c*_ are the variable standard deviation for the normal non-faller group, and clinical faller group, respectively; *μ*_*n*_ and *μ*_*c*_ are the variable mean for the normal non-faller group, and clinical faller group, respectively [31]. This method is considered to be more appropriate than “two standard deviations from the mean” as a clinically meaningful difference because two standard deviations can be too stringent or too lenient, depending on whether the clinical or normal group is used to establish the cut-off score [32].

For Receiver-Operator Characteristic (ROC) curves the predictive value was based on area-under-curve, accuracy, sensitivity, and specificity. Area-under-curve values near one indicate a higher chance of correct classification, whereas values near zero indicate a higher chance of incorrect classification. This method allows the user to choose a cut-off score that corresponds to a desired sensitivity or specificity. A cut-off score with at least 80% sensitivity was selected because correctly identifying fallers in a clinical setting would be more important than minimizing false positives.

A linear discriminant function was based on all variables that showed a significant difference between PNF and PF, and between PNF and PMF. Discriminant analysis is similar to logistic regression in that both provide a linear classification between the two outcomes (faller and non-faller groups) and tend to provide similar results [33,34] when normality assumptions are not overly violated, which would occur with qualitative or categorical independent variables [33]. The cut-off score was the mean value between the discriminant function group centroid values.

## Results

### Differences between eyes open and closed trials

Eyes-closed results were significantly greater than for eyes-open, for PNF, PF, and PSF groups, for AP range of CoP motion, AP RMS, AP and ML CoP velocities, and CoP VSM velocity (Table 2). For PNF, the largest percent increase (eyes open to eyes closed) was 104% for AP velocity, followed by AP range, VSM velocity, AP RMS, and ML velocity. For PAF, the largest percent increase was 118% for AP velocity, followed by VSM velocity, AP range, AP RMS, and ML velocity. For PF, the largest percent increase was 118% for AP velocity, followed by VSM velocity, AP range, AP RMS, and ML velocity. For PSF, the largest percent increase was 99% for AP velocity, followed by VSM velocity, AP range, ML velocity, and AP RMS. For PMF, all variables were significantly greater for eyes closed than eyes open (Table 2). The largest percent increase was 186% for AP velocity, followed by VSM velocity, AP range, AP RMS, ML velocity, ML RMS, and ML range. Comparison and percent increases between eyes-open and eyes-closed posturography results for RNF and RF were reported previously in [22].

**Table 2:** Mean, standard deviation, and p-value between eyes open and eyes closed conditions and percent increase from eyes open to eyes closed conditions.

Measures	Eyes Open	Eyes Closed	*p* value	Percent Increase (%)
*Prospective Non-Faller*				
CoP Range, AP (mm)	21.42 ± 7.24	37.72 ± 12.99	**<0.001**	90.3 ± 78.7
CoP Range, ML (mm)	14.98 ± 9.70	15.58 ± 7.09	0.662	24.7 ± 60.6
CoP RMS, AP (mm)	4.12 ± 1.22	6.91 ± 2.35	**<0.001**	76.5 ± 64.5
CoP RMS, ML (mm)	2.80 ± 1.80	2.86 ± 1.20	0.810	20.1 ± 54.9
CoP Velocity, AP (mm/s)	7.53 ± 1.93	15.11 ± 5.59	**<0.001**	104.1 ± 65.1
CoP Velocity, ML (mm/s)	4.57 ± 1.57	5.83 ± 2.01	**<0.001**	33.7 ± 45.3
CoP Velocity, VSM (mm/s)	9.70 ± 2.34	17.26 ± 6.04	**<0.001**	81.3 ± 56.9
*Prospective All Fallers*				
CoP Range, AP (mm)	22.04 ± 5.81	34.21 ± 14.84	**<0.001**	57.4 ± 50.9
CoP Range, ML (mm)	13.10 ± 8.90	14.92 ± 9.58	**0.031**	24.5 ± 50.7
CoP RMS, AP (mm)	4.48 ± 1.21	6.57 ± 2.55	**<0.001**	50.8 ± 47.8
CoP RMS, ML (mm)	2.55 ± 1.77	2.84 ± 1.97	0.156	21.8 ± 54.3
CoP Velocity, AP (mm/s)	7.75 ± 2.15	17.76 ± 13.40	**<0.001**	118.4 ± 99.5
CoP Velocity, ML (mm/s)	4.62 ± 1.69	6.86 ± 5.37	**<0.001**	42.0 ± 56.2
CoP Velocity, VSM (mm/s)	9.86 ± 2.77	20.30 ± 15.05	**<0.001**	99.1 ± 100.6
*Prospective Faller*				
CoP Range, AP (mm)	22.86 ± 5.47	34.00 ± 12.37	**<0.001**	52.6 ± 53.7
CoP Range, ML (mm)	13.43 ± 9.97	14.32 ± 5.77	0.665	21.9 ± 47.9
CoP RMS, AP (mm)	4.65 ± 1.25	6.51 ± 2.03	**<0.001**	45.7 ± 47.6
CoP RMS, ML (mm)	2.65 ± 1.94	2.72 ± 1.41	0.864	18.3 ± 57.3
CoP Velocity, AP (mm/s)	7.75 ± 1.66	17.03 ± 8.39	**<0.001**	117.9 ± 97.4
CoP Velocity, ML (mm/s)	4.63 ± 1.67	6.74 ± 4.53	**0.007**	42.9 ± 56.6
CoP Velocity, VSM (mm/s)	9.84 ± 2.32	19.58 ± 9.82	**<0.001**	101.5 ± 106.6
*Prospective Single-Faller*				
CoP Range, AP (mm)	22.97 ± 4.99	31.85 ± 11.12	**0.001**	40.3 ± 44.2
CoP Range, ML (mm)	14.24 ± 11.15	14.11 ± 6.33	0.338	15.7 ± 50.5
CoP RMS, AP (mm)	4.71 ± 1.34	6.00 ± 1.72	**0.005**	32.4 ± 36.8
CoP RMS, ML (mm)	2.80 ± 2.17	2.63 ± 1.52	0.745	9.8 ± 58.0
CoP Velocity, AP (mm/s)	7.78 ± 1.74	15.66 ± 7.13	**<0.001**	99.3 ± 71.1
CoP Velocity, ML (mm/s)	4.73 ± 1.84	6.73 ± 5.06	**0.040**	38.5 ± 60.4
CoP Velocity, VSM (mm/s)	10.02 ± 2.39	18.30 ± 9.25	**<0.001**	80.1 ± 66.6
*Prospective Multi-Faller*				
CoP Range, AP (mm)	22.48 ± 7.52	41.91 ± 14.55	**0.046**	97.6 ± 65.5
CoP Range, ML (mm)	10.49 ± 1.22	15.09 ± 3.25	**0.046**	44.7 ± 30.1
CoP RMS, AP (mm)	4.44 ± 0.96	8.38 ± 2.12	**0.028**	94.7 ± 53.7
CoP RMS, ML (mm)	2.08 ± 0.41	3.07 ± 0.99	**0.028**	49.4 ± 46.3
CoP Velocity, AP (mm/s)	7.67 ± 1.43	22.06 ± 11.31	**0.028**	186.0 ± 151.4
CoP Velocity, ML (mm/s)	4.28 ± 0.86	6.77 ± 1.89	**0.046**	59.2 ± 39.4
CoP Velocity, VSM (mm/s)	9.21 ± 2.12	24.27 ± 11.31	**0.046**	179.9 ± 182.7

### Differences between fallers and non-fallers

RQ for AP range and AP RMS were significantly greater for PNF than PAF (Table 3). RQ cut-off scores for PAF based on RQ AP range and RQ AP RMS achieved 55.1–61.8% accuracy, 61.9–81.0% sensitivity, and 31.9–57.4% specificity (Table 4). The RQ for AP range clinical cut-off score achieved the best accuracy and specificity results and ROC cut-off score achieved the best sensitivity results for discriminating between PAF and PNF (Table 4).

**Table 3:** Romberg Quotient mean, standard deviation, and p-values for comparisons between faller groups and prospective non-fallers (PNF).

	PNF	PAF	*p*	PF	*p*	PSF	*p*	PMF	*p*
CoP Range, AP	1.90 ± 0.79	1.57 ± 0.51	**0.021**	1.53 ± 0.54	**0.028**	1.40 ± 0.44	**0.007**	1.98 ± 0.65	0.651
CoP Range, ML	1.25 ± 0.61	1.25 ± 0.51	0.718	1.22 ± 0.48	0.827	1.16 ± 0.50	0.522	1.45 ± 0.30	0.213
CoP RMS, AP	1.77 ± 0.65	1.51 ± 0.48	**0.037**	1.46 ± 0.48	**0.021**	1.32 ± 0.37	**0.004**	1.95 ± 0.54	0.401
CoP RMS, ML	1.20 ± 0.55	1.22 ± 0.54	0.824	1.18 ± 0.57	0.892	1.10 ± 0.58	0.487	1.49 ± 0.46	0.197
CoP Velocity, AP	2.04 ± 0.65	2.18 ± 0.99	0.915	2.18 ± 0.97	0.510	1.99 ± 0.71	0.790	2.86 ± 1.51	**0.019**
CoP Velocity, ML	1.34 ± 0.45	1.42 ± 0.56	0.675	1.43 ± 0.57	0.467	1.38 ± 0.60	0.743	1.59 ± 0.39	0.187
CoP Velocity, VSM	1.81 ± 0.57	1.99 ± 1.01	0.993	2.02 ± 1.07	0.360	1.80 ± 0.67	0.944	2.80 ± 1.83	**0.006**

Prospective Non-Fallers (PNF), Prospective All Fallers (PAF), Prospective Faller (PF), Prospective Single-Fallers (PSF), Prospective Multi-Fallers (PMF)

**Table 4:** Prospective all fallers (PAF) clinical, ROC, and discriminant function cut-off scores (classified as faller for scores less than the cut-off score).

Method	Measure	Cut-Off Score	Accuracy (%)	Sensitivity (%)	Specificity (%)
Clinical	RQ CoP Range, AP	1.70	61.8	66.7	57.4
	RQ CoP RMS, AP	1.62	57.3	61.9	53.2
ROC	RQ CoP Range, AP (AUC = 0.614)	2.07	57.3	81.0	36.2
	RQ CoP RMS, AP (AUC = 0.616)	2.01	55.1	81.0	31.9
Discriminant Function	-2.799 + 1.092 x RQAPRange + 0.542 x RQAPRMS	-0.014	56.8	64.3	50.0

RQAPRange: RQ CoP Range, AP; RQAPRMS: RQ CoP RMS, AP

RQ for AP range and AP RMS were significantly greater for PNF than PF (Table 3). RQ cut-off scores for PF based on RQ AP range and RQ AP RMS achieved 56.0–62.7% accuracy, 60.7–82.1% sensitivity, and 40.4–57.4% specificity (Table 5). The RQ for AP range clinical cut-off score achieved the best accuracy and specificity results and ROC cut-off score achieved the best sensitivity results for discriminating between PF and PNF (Table 5).

**Table 5:** Prospective faller (PF) clinical, ROC, and discriminant function cut-off scores (classified as faller for scores less than the cut-off score).

Method	Measure	Cut-Off Score	Accuracy (%)	Sensitivity (%)	Specificity (%)
Clinical	RQ CoP Range, AP	1.68	62.7	71.4	57.4
	RQ CoP RMS, AP	1.59	57.3	60.7	55.3
ROC	RQ CoP Range, AP (AUC = 0.633)	1.96	57.3	82.1	42.6
	RQ CoP RMS, AP (AUC = 0.640)	1.81	56.0	82.1	40.4
Discriminant Function	-2.779 + 0.812 x RQAPRange + 0.817 x RQAPRMS	-0.071	57.3	67.9	51.1

RQAPRange: RQ CoP Range, AP; RQAPRMS: RQ CoP RMS, AP

RQ for AP range and AP RMS were significantly greater for PNF than PSF (Table 3). RQ cut-off scores for PSF based on RQ AP range and RQ AP RMS achieved 60.9–66.7% accuracy, 63.6–81.8% sensitivity, and 53.2–61.7% specificity (Table 6). The RQ for AP range ROC cut-off score achieved the best accuracy and sensitivity results and the clinical cut-off score achieved the best specificity results for discriminating between PSF and PNF (Table 6).

**Table 6:** Prospective single faller (PSF) clinical, ROC, and discriminant function cut-off scores (classified as faller for scores less than the cut-off score).

Method	Measure	Cut-Off Score	Accuracy (%)	Sensitivity (%)	Specificity (%)
Clinical	RQ CoP Range, AP	1.58	65.2	72.7	61.7
	RQ CoP RMS, AP	1.48	60.9	63.6	59.6
ROC	RQ CoP Range, AP (AUC = 0.686)	1.64	66.7	81.8	59.6
	RQ CoP RMS, AP (AUC = 0.708)	1.63	62.3	81.8	53.2
Discriminant Function	-2.863 + 0.524 x RQAPRange + 1.200 x RQAPRMS	-0.144	62.3	77.3	55.3

RQAPRange: RQ CoP Range, AP; RQAPRMS: RQ CoP RMS, AP

PMF eyes closed AP velocity (p = 0.015) and eyes closed VSM (p = 0.020) were significantly greater than PNF. The Romberg Quotients for AP velocity and VSM were also significantly greater for PMF compared to PNF (Table 3). PMF cut-off scores for eyes closed AP velocity, eyes closed VSM, RQ for AP velocity, and RQ for VSM velocity achieved 45.3–84.9% accuracy, 50.0–83.3% sensitivity, and 40.4–89.4% specificity (Table 7). Discriminant function achieved the best accuracy and specificity results (Table 7). ROC cut-off scores achieved the best sensitivity results for discriminating between PMF and PNF (Table 7).

**Table 7:** Prospective multiple faller (PMF) clinical, ROC, and discriminant function cut-off scores (classified as faller for scores greater than the cut-off score).

Method	Measure	Cut-Off Score	Accuracy (%)	Sensitivity (%)	Specificity (%)
Clinical	CoP Velocity, AP, Eyes Closed (mm/s)	17.41	69.8	50.0	72.3
	CoP Velocity, VSM, Eyes Closed (mm/s)	19.70	67.9	50.0	70.2
	RQ CoP Velocity, AP	2.29	71.7	50.0	74.5
	RQ CoP Velocity, VSM	2.05	69.8	50.0	72.3
ROC	CoP Velocity, AP, Eyes Closed (mm/s) (AUC = 0.688)	13.78	49.1	83.3	44.7
	CoP Velocity, VSM, Eyes Closed (mm/s) (AUC = 0.691)	15.34	47.2	83.3	42.6
	RQ CoP Velocity, AP (AUC = 0.660)	1.83	45.3	83.3	40.4
	RQ CoP Velocity, VSM (AUC = 0.670)	1.69	45.3	83.3	40.4
Discriminant Function	-1.481 + 0.146 x APVelEC—0.114 x VSMVelEC—2.027 x RQAPVel + 2.877 x RQVSMVel	0.541	84.9	50.0	89.4

APVelEC: CoP velocity, AP, eyes closed; VSMVelEC: CoP velocity, VSM, eyes closed; RQAPVel: RQ CoP velocity, AP; RQVSMVel: RQ CoP velocity, VSM

No significant differences were found between RF and RNF for eyes open, eyes closed, and RQ (Table 3) posturography values.

Based on these results, the recommended cut off score for classifying fallers and non-fallers was 1.68 for RQ of AP CoP range (Table 5). The cut off scores for classifying multi-fallers (Table 7) was 0.541 based on a discriminant function that includes eyes closed AP velocity, eyes closed VSM velocity, RQ of AP velocity, and RQ of VSM velocity (-1.481 + 0.146 x APVelEC—0.114 x VSMVelEC—2.027 x RQAPVel + 2.877 x RQVSMVel).

## Discussion

Static posturography measures in this study discriminated between elderly fallers and non-fallers, with RQ and AP measures being particularly relevant for fall risk classification. Since multi-faller cut-off score classifications had high accuracy, sensitivity, or specificity, an eyes open: eyes closed static posturography assessment can be considered a viable screening tool for older people at risk of falling.

For all participants, measures sensitive to AP motion increased when visual input was removed, with the largest percent increases for PMF. This suggests that older adults have an increased reliance on visual input for postural control, particularly older adults at increased risk of multiple falls. For PAF, PF and PNF, percent increases from eyes open to eyes closed were inconsistent, with PAF and PF having greater percent increases in AP velocity (PAF: 118%. PF: 118%, PNF: 104%) but smaller percent increases in AP range (PAF: 57%, PF: 53%, PNF: 90%) and RMS (PAF: 51%, PF: 46%, PNF: 77%). These differences in distance (range, RMS) and velocity may be because PNF might be able to tolerate a larger range of CoP movements, allowing them to better withstand potentially fall-inducing perturbations [16]. Conversely, PAF and PF may have a lower tolerance, to compensate for poorer postural control, requiring increased AP velocities compared to PNF to maintain CoP within a smaller area of stability. PMF may be unable to maintain a smaller range of CoP movements because of postural control issues that result in greater increases in range, RMS, and velocity. Greater AP CoP movement with eyes closed was also found within RF and RNF groups, but AP CoP measures could not discriminate between RF and RNF, as reported in [22].

For ML measures, CoP velocity increased with eyes closed for PNF, PAF, PF, and PSF. However, all ML measures for PMF increased with eyes closed. These results further support the premise that PMF have poorer postural control than PNF and PSF. Since significant increases in ML range only occurred for PAF and PMF and significant increases in ML RMS only occurred for PMF, ML balance control assessment with eyes closed may only be important for evaluating the risk of multiple falls (i.e., people at higher fall risk).

Romberg Quotient was the best measure for differentiating between PAF and PNF, between PF and PNF and between PSF and PNF, with significant differences for RQ AP range and RQ AP RMS. PMF had greater RQ AP velocity and RQ VSM velocity than PNF. These results highlighted the importance of testing postural balance with and without visual input and calculating RQ to give the clearest indication of fall risk, for both single and multi-fallers, in an older adult population.

AP and VSM measures were better at discriminating between faller and non-faller populations compared to ML measures, although ML measures performed well in the review paper by Piirtola and Era [4]. Associations between AP posturography measures and faller status have also been previously reported in the literature [9–11,14]. In this study, the association between AP outcomes and faller status could be due to the less challenging (i.e., non-foam surface) posturography assessment that may invoke an ankle-based control strategy with predominant AP movements, instead of a hip-based control strategy with predominant ML movements, as suggested by Maranesi et al. [10]. Another explanation could be the more natural stance width when using two Wii Balance Boards instead of narrow-based stance used in other studies [4], as suggested by Merlo et al. [11].

Differences were not found between RF and RNF in our previous, preliminary work [22] and in the additional RQ analysis presented in this paper. In the current paper, retrospective and prospective fall occurrence analyses were performed on the same population; however, retrospective fallers were excluded from the prospective analysis because retrospective fallers were already identified as at risk of falling based on their fall history. Initial analysis with retrospective fall occurrence data provided a preliminary evaluation of static posturography and fall prediction. However, retrospective fall occurrence is limited by inaccurate recall of falls and movement patterns may change after a fall to increase stability or reduce fear of falling [35]. Furthermore, only two retrospective fallers were multi-fallers (8.3% of RF) [22], whereas six prospective fallers were multi-fallers (21.4% of PF). The larger proportion of multi-fallers, who likely had greater balance issues compared to one-time fallers, in the PF group compared to the RF group may partially explain the presence of static posturography differences between faller and non-faller groups in the prospective analysis and the lack of static posturography differences between faller and non-faller groups in the retrospective analysis. The larger number of multi-fallers in the prospective analysis also allowed a multi-faller subgroup analysis, which could not be performed in the retrospective analysis. In the previous preliminary analysis [22], PF were included in the RNF group because they had not yet experienced a fall (i.e., the 6-month follow-up period had not yet occurred at the time of that preliminary study). Since prospective fallers likely had balance issues that contributed to their prospective fall, these PF in the RNF group may have masked balance differences between RF and RNF and may explain the lack of static posturography differences in the initial retrospective analysis [22].

The clinical cut-off score achieved the best results for PAF and PF classification and the discriminant function cut-off score achieved the best results for PMF classification, in terms of accuracy and specificity. For PSF classification, the ROC cut-off score achieved the best accuracy and sensitivity and the clinical cut-off score achieved the best specificity. The discriminant function cut-off score performed better for PMF classification possibly because four significant variables were included in the analysis, whereas PAF, PF and PSF classifications had only two significant variables. With a larger number of relevant variables, the discriminant function cut-off scores outperformed clinical and ROC cut-off scores, which were based on only one variable. However, the clinical and ROC cut-off scores outperformed discriminant function cut-off scores when only two significant variables were used for PAF, PF and PSF classifications, which may be partly due to the consideration of variance in the clinical calculation. PAF, PF and PSF cut-off scores produced moderate faller classification results that were comparable to the literature (i.e., 62–67% accuracy, 67–82% sensitivity, 57–60% specificity versus Topper et al. [17] with 65% accuracy, 78% sensitivity, 46% specificity). For PMF, a discriminant function-based cut-off score achieved good fall risk classification results (85% accuracy, 50% sensitivity, and 89% specificity). The PMF cut-off score classification outperformed PAF, PF and PSF cut-off score classification in terms of accuracy and specificity. Identifying and classifying PSF can be challenging because some one-time fallers may have fallen due to environmental causes (e.g., unexpected obstacle, icy conditions, etc.) and may have relatively good balance compared to other fallers. Including fallers who fell due primarily to non-biomechanical reasons could increase classification difficulty and could decrease classification accuracy. Practical application of these results would support use of the PF method to identify people at risk of falling once and then applying the PMF method to identify people at risk of falling more than once.

While clinical and discriminant function cut-off scores achieved the best results for PAF, PF and PMF, the ROC cut-off score achieved the best sensitivity results because a preferred sensitivity level can be set with the ROC method. It is therefore important to consider the classification goals when choosing a cut-off score method, because the ROC would be preferable when it is important to not misclassify fallers as non-fallers.

With only six multi-fallers, the multi-faller sensitivity results for the determined cut-off scores were limited to one of seven levels (0%, 17%, 33%, 50%, 67%, 83%, and 100%). More precise predictive sensitivity could be determined with a larger sample of multi-fallers. The PMF population also weighed more than the other study participants, which could have influenced the posturography results [36]. Medical information such as disease status, alcoholism, etc. was not collected, and therefore no evaluation of the impact of these factors on posturography results could be performed. While longer or repeated posturography trials may be preferred for postural stability assessments, a single, short trial was used in this study for maximal clinical utility. Future research is recommended to evaluate test-retest reliability of this fall risk assessment approach.

## Conclusions

Static posturography measures can discriminate between elderly fallers and non-fallers. Differences were found between fallers and non-fallers for RQ AP range and RQ AP RMS and between multi-fallers and non-fallers for eyes closed AP velocity, eyes closed VSM velocity, RQ AP, and RQ VSM velocity, suggesting that RQ calculations are particularly relevant for elderly fall risk assessments. Cut-off scores based on posturography measures were clinically viable for multi-faller classification and provided better accuracy than for single-faller classification. RQ CoP AP range with a 1.64 cut-off score could be used to screen for older people who may fall once. PMF classification with a discriminant function (-1.481 + 0.146 x *Eyes Closed AP Velocity*—0.114 x *Eyes Closed Vector Sum Magnitude Velocity*—2.027 x *RQ AP Velocity* + 2.877 x *RQ Vector Sum Magnitude Velocity*) and cut-off score of 0.541 could be used as a viable screening tool for older people at risk of multiple falls.

## Supporting information

S1 File Fall information form. Data collection form for each fall.
(PDF)
