# Supplementary material for: Elderly fall risk prediction using static posturography
Source: PLoS One. 2017 Feb 21;12(2):e0172398. doi: 10.1371/journal.pone.0172398 (PMC5319679; doi:10.1371/journal.pone.0172398)
Supplement: S1 File — Data collection form for each fall. (PDF) [file pone.0172398.s001.pdf]

Detailed Fall Information

Date of Fall: ________________

Please check the boxes as appropriate:

When did this happen?

Morning ☐ Afternoon ☐

Evening ☐ Night ☐

Where did this happen?

Outside ☐ At Home ☐

At a Friend's ☐ Other Indoor Location ☐

Other: _______________________________

Were you using your walker/cane/orthotics/other walking aid when the fall/near miss happened?

Yes ☐ No ☐ I don't have one ☐

What do you think caused you to fall or almost fall?

Alcohol consumption in the last 12 hours: _________

Medications used in the last 12 hours: ____________
